# Management of Hepatocellular Carcinoma during the COVID-19 Pandemic - São Paulo Clínicas Liver Cancer Group Multidisciplinary Consensus Statement

**DOI:** 10.6061/clinics/2020/e2192

**Published:** 2020-11-10

**Authors:** Aline Lopes Chagas, Leonardo Gomes da Fonseca, Fabricio Ferreira Coelho, Lisa Rodrigues da Cunha Saud, Edson Abdala, Wellington Andraus, Lucas Fiore, Airton Mota Moreira, Marcos Roberto Menezes, Francisco César Carnevale, Claudia Megumi Tani, Regiane S.S.M Alencar, Luiz Augusto Carneiro D'Albuquerque, Paulo Herman, Flair José Carrilho

**Affiliations:** I Departamento de Gastroenterologia, Divisao de Gastroenterologia e Hepatologia Clinica, Hospital das Clinicas (HCFMUSP), Faculdade de Medicina, Universidade de Sao Paulo, Sao Paulo, SP, BR; II Instituto do Cancer do Estado de Sao Paulo (ICESP), Hospital das Clinicas (HCFMUSP), Faculdade de Medicina, Universidade de Sao Paulo, Sao Paulo, SP, BR; III São Paulo Clínicas Liver Cancer Group, Hospital das Clinicas (HCFMUSP), Faculdade de Medicina, Universidade de Sao Paulo, Sao Paulo, SP, BR; IV Departamento de Gastroenterologia, Divisao de Cirurgia do Aparelho Digestivo, Hospital das Clinicas (HCFMUSP), Faculdade de Medicina, Universidade de Sao Paulo, Sao Paulo, SP, BR; V Departamento de Gastroenterologia, Divisao de Transplante de Orgaos do Aparelho Digestivo, Hospital das Clinicas (HCFMUSP), Faculdade de Medicina, Universidade de Sao Paulo, Sao Paulo, SP, BR; VI Departamento de Radiologia e Oncologia, Faculdade de Medicina (FMUSP), Universidade de Sao Paulo, Sao Paulo, SP, BR

**Keywords:** Hepatocellular Carcinoma, Hepatocellular Carcinoma/Therapy, COVID-19, Clinical Practice Guideline

## Abstract

More than 18 million people in 188 countries have been diagnosed as having coronavirus disease (COVID-19), and COVID-19 has been responsible for more than 600,000 deaths worldwide. Brazil is now the second most affected country globally. Faced with this scenario, various public health measures and changes in the daily routines of hospitals were implemented to stop the pandemic. Patients with hepatocellular carcinoma (HCC) are at an increased risk for severe COVID-19 as they present with two major diseases: cancer and concomitant chronic liver disease. The COVID-19 pandemic can significantly impact the management of HCC patients from diagnosis to treatment strategies. These patients need special attention and assistance at this time, especially since treatment for tumors cannot be delayed in most cases. The aim of this guideline was to standardize the management of HCC patients during the COVID-19 pandemic. This document was developed, on the basis of the best evidence available, by a multidisciplinary team from Instituto do Câncer do Estado de São Paulo (ICESP), and Instituto Central of the Hospital das Clínicas da Universidade de São Paulo (HC-FMUSP), which are members of the São Paulo Clínicas Liver Cancer Group.

## INTRODUCTION

Coronavirus disease (COVID-19) is caused by severe acute respiratory syndrome coronavirus 2 (SARS-CoV-2), a novel coronavirus that was first reported in December 2019 in Wuhan, China (1). The number of cases has increased exponentially worldwide, and COVID-19 has now been declared a pandemic, with more than 18 million people in 188 countries having been diagnosed with COVID-19 and more than 600,000 deaths from COVID-19 (2).

Brazil also experienced a significant increase in the number of cases. By August 4, the country had 2,750,318 documented cases and 94,665 confirmed deaths, making it the second most affected country worldwide (2,3). Faced with this scenario, public health measures, such as the implementation of social distancing and changes in the daily routines of hospitals, were implemented to curtail the pandemic (4). Within the Hospital das Clínicas da Universidade de São Paulo (HC-FMUSP) complex, structural changes, as well as changes in patient management, were implemented not only to provide assistance to patients with COVID-19 but also to provide adequate support to the rest of the patients in the complex (4).

Patients with hepatocellular carcinoma (HCC) need special attention and assistance at this time especially because tumor treatment, in most cases, cannot be delayed and adequate follow-up is essential for the early detection of recurrence. Furthermore, most HCC patients have concomitant cirrhosis and thus are afflicted with two comorbidities that potentially increase their risk for severe COVID-19 (5).

This document was developed by a multidisciplinary team from Instituto do Câncer do Estado de São Paulo and Instituto Central of the HC-FMUSP, both of which are members of the São Paulo Clínicas Liver Cancer Group. The aim of this guideline was to standardize the management of HCC patients during the COVID-19 pandemic on the basis of the best available evidence.

### Risk of COVID-19 in cancer patients

Some reports have suggested that patients with cancer may be at an increased risk for SARS-CoV-2 infection (6-9). In a systematic review of 11 studies, the prevalence of previously diagnosed cancer in patients with COVID-19 was reported at 2% (95%CI, 2%-3%) (8). Another systematic review showed that 0.92% of nearly 77,000 hospitalized patients with COVID-19 had previously been diagnosed with cancer (9).

Patients undergoing cancer treatments experience concomitant immunosuppression and are thus expected to have higher morbidity and mortality rates from COVID-19 than those in others. In addition, most of these patients are elderly and have associated comorbidities which increases the risk of developing more severe SARS-CoV-2 infection (5,6). However, the evidence supporting this inference is limited to observational series published in the past few months (6,7,10-12). In a study reported by Deng et al. (10) on 44,672 patients with COVID-19, patients with cancer had a significantly higher risk of death than did those without cancer (RR=2.93, 95%CI 1.34-6.41, *p*=0.006). Mehta et al. (11) evaluated 218 patients with both cancer and COVID-19 and demonstrated a higher mortality rate in patients with cancer (28%) than in those without (14%). A recent report on 928 patients with both cancer and COVID-19 from the “COVID-19 and Cancer Consortium database” showed that increased age, male sex, smoking status, number of comorbidities, Eastern Cooperative Oncology Group performance status of 2 or higher, and active cancer were independent factors associated with increased 30-day mortality (13). 

### Impact of COVID-19 in the management of HCC patients

To date, there are no data on the outcomes of patients with COVID-19 and HCC. However, these patients are likely at an increased risk for severe COVID-19 as most of them present with two major diseases: cancer and concomitant chronic liver disease. In fact, the COVID-19 pandemic can significantly impact the management of HCC patients via the following (5,14):

- Having a negative impact on HCC screening programs that use abdominal ultrasonography (US) in patients with cirrhosis and other high-risk groups, thereby leading to delays in diagnosis.
- Limiting access to imaging examinations, such as computed tomography (CT) and magnetic resonance imaging (MRI), and procedures, such as liver biopsy, thereby leading to delays in diagnosis.
- Restricting access to Hepatology referral centers for the diagnosis and treatment of HCC.
- Decreasing the availability of human resources to care for HCC patients.
- Decreasing the availability of anesthesiologists to perform surgeries and procedures such as percutaneous ablative therapies.
- Reducing the availability of hospital beds, intensive care units (ICU), and operating rooms for treatment and post-operative care.
- Increasing treatment-related morbidity and mortality in patients with cirrhosis and cancer because of their increased risk of developing severe COVID-19.

In the face of such a challenging scenario, it became essential to review the guidelines for HCC diagnosis and treatment and modify them to fit the current situation. Here, we describe the recommendations for HCC management according to available data and the most recent guidelines, which were discussed by a multidisciplinary team and adapted to our country's requirements (5,).

## HCC SURVEILLANCE AND DIAGNOSIS DURING THE COVID-19 PANDEMIC

Early diagnosis of HCC allows treatment to be initiated with curative intent and has a great impact on prognosis. The following are the recommendations for the screening and diagnosis of HCC during the pandemic (5,):

- Screening for HCC using US every 6 months in patients who are at a high risk of developing this tumor (*e.g*., cirrhosis, hepatitis B) should be continued; however, a 2-month delay may be acceptable during the pandemic.
- The first medical consult of recently diagnosed patients or those with a suspected HCC nodule should not be postponed and should be made in person.
- Imaging tests (CT or MRI) or procedures that are necessary for diagnosing HCC in patients with suspicious nodules on US should not be postponed and should be performed as soon as possible.
- Cases that present with undetermined nodules on imaging examinations (CT/MRI) should be discussed in a multidisciplinary web meeting to define a appropriate method to confirm or rule out HCC. This meeting should consider the risks and benefits of performing nodule biopsy, MRI with hepatobiliary contrast, or follow-up imaging examinations depending on the degree of suspicion.

## HCC TREATMENT DURING THE COVID-19 PANDEMIC

The management of HCC during the pandemic has become a major challenge. The Barcelona Clinic Liver Cancer (BCLC) staging system is usually recommended for clinical staging and therapeutic guidance (17). However, during the pandemic, the risks and benefits of HCC treatment should be individualized and factors like tumor stage, liver function, age, comorbidities, local availability of resources, and the risk of SARS-CoV-2 infection should be considered (5,).

### General recommendations

- Tumor treatment should not be delayed whenever possible to avoid disease progression and prevent negative consequences in terms of patient survival.
- Patients with the highest chance for cure as well as those near the top of the liver transplant list should be prioritized for treatment.
- When a “bridge treatment” is chosen, alpha-fetoprotein monitoring and imaging examinations should be performed so that patients are not dropped off the liver transplantation (LT) list and their chances of availing other curative therapies are retained, such as surgery or radiofrequency ablation (RFA).
- In elderly patients (age >75 years), patients with comorbidities, and patients with borderline liver function, the risks and benefits of HCC treatment should be assessed; the feasibility of switching to a less invasive treatment or delaying treatment and opting for rigorous imaging monitoring should be considered.
- Therapeutic decisions, whenever possible, should be discussed in online multidisciplinary meetings especially, for patients who have a high risk for liver decompensation or severe COVID-19.
- Deviations from “standard” treatment should preferably be discussed in online multidisciplinary meetings. The therapeutic decision as well as the associated risks and benefits should be discussed with the patient and properly documented for legal purposes.

### Surgical treatment of HCC during the COVID-19 pandemic

#### Standard treatment indications

The indications for surgical resection are the same as those during the pre-pandemic period and are as follows (18-21):

- In patients without cirrhosis: solitary or oligonodular HCCs.
- In patients with chronic liver disease: solitary tumors (regardless of size), preserved liver function (Child-Pugh A), and absent or mild portal hypertension (small-caliber esophageal varices and platelets >100,000/mm^3^).

### General management of surgical patients

The recommendations for surgical management during the pandemic are aimed at reducing the risk of SARS-CoV-2 infection, which can cause increased morbidity and mortality in the perioperative period (5,22,23).

- Patients should be hospitalized in “COVID-19-free” areas when possible.
- Patients should be advised to undergo social isolation for 14 days before the procedure.
- Patients should be tested for SARS-CoV-2 72 hours before the procedure with reverse transcription-polymerase chain reaction (RT-PCR).
- Given the high rate of false-negative results with laboratory tests, performing a chest CT scan before the procedure is recommended.
- If preoperative testing is not possible, consider the patient as infected.
- If the results of the RT-PCR and/or chest CT are suggestive of SARS-CoV-2 infection, consider postponing the procedure.

### Recommendations during the COVID-19 pandemic

- Select patients who do not have comorbidities that can increase the risk of developing severe forms of COVID-19.
- In patients without liver disease or severe comorbidities and who have single tumors or oligonodular disease, consider the feasibility of surgery.
- Among patients with liver disease, select those with a lower risk of developing liver function decompensation (Child-Pugh A without clinically significant portal hypertension).
- In patients with very early (BCLC-0) or early (BCLC-A) HCC with solitary nodules ≤3 cm in size, RFA can be considered a treatment option.
- Patients with chronic liver disease and solitary tumors that are >3 cm in size:

○ Consider performing surgery for patients who have preserved liver function, have no clinically significant portal hypertension or other comorbidities, are young, and present with favorable locations for resection (19,21).

○ In patients who have tumors in locations unfavorable for surgery, low hepatic functional reserve, comorbidities, and/or advanced age, consider using other treatments to serve as a “bridge” for surgery or as definitive management.

### Treatment options during the COVID-19 pandemic

- Radiofrequency ablation can be performed in patients with very early (BCLC-0) or early (BCLC-A) HCC and have solitary nodules ≤3 cm in size.
- Transarterial chemoembolization/embolization (TACE/TAE): can be performed in patients with solitary nodules >3 cm in size as local disease control or as a “bridge treatment” to surgery.
- Stereotactic body radiation therapy (SBRT): can be considered in patients who have contraindications to RFA or TACE/TAE.
- Systemic therapy: may be used as a “bridge therapy” for surgery in patients with contraindications to other treatments.
- In select cases, strict follow-up with imaging may be carried out.

### Liver Transplantation for HCC during the COVID-19 pandemic

The indications for LT are also similar to those before the pandemic (18-20).

### Standard treatment indications

- Liver Transplantation is the treatment of choice for patients with early HCC (BCLC-A) and impaired liver function (Child-Pugh B/C), clinically significant portal hypertension, and those with early HCC who are not candidates for resection (18,19).
- The selection criteria for LT in HCC that was adopted by Brazil is an expanded version of the classic Milan Criteria. In the so-called “Brazilian Milan Criteria,” patients are eligible for an “HCC exception score” if they present with one nodule 2-5 cm in size or up to three nodules 2-3 cm in size, plus any number of nodules <2 cm in size (19).

### Potential impacts of COVID-19 on LT

In addition to the possible impacts of COVID-19 that are common to other treatments, LT may be affected by the pandemic in other aspects (5,14,15):

- Risk of SARS-CoV-2 infection from donors.
- Reduced access to the evaluation process for inclusion in the LT waiting list.
- Increased time in the LT waiting list because of a reduction in the number of donors and number of LTs performed, as well as an increased risk of drop-out because of tumor progression.

### General management of LT patients

In addition to the recommendations already mentioned in the previous section for surgical treatment, the following are suggestions regarding LT during the pandemic (5,14,15):

- Systematically test LT candidates (recipients) for SARS-CoV-2 infection using RT-PCR and chest CT before the procedure.
- Select liver donors from patients with the lowest risk of SARS-CoV-2 infection: patients who are hospitalized in ICUs without suspected or confirmed cases of COVID-19, are negative for SARS-CoV-2 by RT-PCR, and have no suspicious lesions on chest CT scan.

### Recommendations during the COVID-19 pandemic

- It may be necessary to consider temporary suspension of living donor LTs to protect both donors and recipients.
- Consider delaying LT in patients with a complete response to “bridge therapies” and maintain close monitoring with imaging examinations to detect any recurrence.
- Patients with HCC who have significant liver dysfunction and/or viable tumors and have a high risk of losing eligibility for transplantation, especially those who do not respond to “bridge therapies” or present with tumor progression, should remain in the list of LT recipients.
- The risks and benefits of delaying LT should be analyzed on a case-by-case basis and discussed with the patient.
- When faced with the possibility of reduced organ supply and increased time in the liver transplant waiting list, administration of “bridge therapies” with locoregional therapies (RFA, TACE/TAE, or SBRT) should be considered.

### Treatment options during the COVID-19 pandemic

- Radiofrequency ablation can be performed as a “bridge therapy” or as a definitive treatment for patients with solitary nodules ≤3 cm in size.
- Transarterial quimioembolization/embolization as a “bridge therapy”: can be used mainly for patients with solitary tumors that are 3-5 cm in size or for multifocal HCC.
- Stereotactic body radiation therapy may be used as a “bridge therapy” for patients with contraindications to RFA or TACE/TAE.
- Systemic therapy: may be used as a “bridge therapy” for patients with contraindications to other treatments.
- Strict follow-up with imaging can be carried out in selected cases, especially in patients who are not eligible for “bridge therapies,” have severe liver dysfunction (*e.g*., decompensated cirrhosis/Child-Pugh ≥B9), and/or are experiencing physical deterioration.

### Percutaneous ablative therapies during the COVID-19 pandemic

The percutaneous ablative therapies that are most commonly performed in our country are RFA and percutaneous ethanol injection (PEI) (19); the standard indications for their use are maintained during the COVID-19 pandemic (18-20). Some of the characteristics of these procedures, such as decreased invasiveness, lower morbidity rates, and shorter length of hospital stay, make them valuable options during the pandemic (24).

### Standard treatment indications

- Radiofrequency ablation is the treatment of choice for patients with very early (BCLC-0) or early (BCLC-A) HCC who are not candidates for liver resection or LT; this procedure is preferred in patients with tumors that are <3 cm in size.
- In patients with very early HCC (BCLC-0) and solitary tumors that are ≤2 cm deep in the liver parenchyma, RFA can be the first choice of treatment even if these patients are candidates for surgery. In patients with solitary tumors that are 2-3 cm in size and who are candidates for resection, RFA may be considered an alternative to surgery depending on the tumor location and the patient’s clinical status.
- The use of PEI may be recommended in cases of very early (BCLC-0) and early (BCLC-A) HCC when RFA is not technically possible or unavailable, and especially for tumors that are <2 cm in size.
- Radiofrequency ablation and/or PEI may be performed in patients with early HCC (BCLC-A) who are on the LT waiting list as a “bridge therapy.”

### Recommendations during the COVID-19 pandemic

- Select patients who have a low risk of developing complications.
- Select patients with the highest chance of cure (solitary tumors <3 cm).
- Consider performing RFA as an outpatient procedure in selected patients.
- For elderly patients, patients with comorbidities, and patients with solitary tumors that are ≤2 cm in size, PEI can be performed as an outpatient procedure.
- Transarterial quimioembolization should be performed before RFA for patients with solitary tumors that are 3-5 cm in size to reduce the size of the lesion, increase the efficacy of the ablation procedure, and reduce the risk of developing RFA-related complications.

### Treatment options during the COVID-19 pandemic

- Transarterial quimioembolization/embolization can be used for patients with tumors that are 3-5 cm in size, tumors with locations unfavorable for RFA treatment, or as a neoadjuvant treatment to improve outcomes.
- Stereotactic body radiation therapy can be used for patients with contraindications to TACE/TAE.
- Systemic therapy: can be used for patients with contraindications to other treatments.
- Strict follow-up with imaging in selected cases. This option should be considered for patients with BCLC-0 HCC (solitary nodule <2 cm), elderly patients (age >75 years), and/or patients with comorbidities that increase the risk of developing severe forms of COVID-19.

### Imaging-guided transarterial therapies during the COVID-19 pandemic

The traditional indications for performing imaging-guided transarterial therapies are maintained during the COVID-19 pandemic (17-20). Of these therapies, the most widely used in our country are TACE and TAE. Other treatments include drug-eluting bead embolization and transarterial radioembolization (19). Unfortunately, these treatments are not available within the HC-FMUSP complex and thus were not included in this guideline.

Because these procedures are less invasive, do not require general anesthesia, and require only a short hospital stay, they can serve as good alternatives during the pandemic as “bridge therapies” for surgery or transplantation or as definitive treatment (5,14).

### Standard treatment indications

- Patients with intermediate HCC (BCLC-B) and preserved liver function.
- As a “bridge therapy” for patients with early HCC (BCLC-A) who are on the LT waiting list, especially for those with solitary tumors that are 3-5 cm in size or with two or three nodules.
- Patients with early HCC (BCLC-A) who have contraindications to surgical treatment or percutaneous ablative therapies.

### Recommendations during the COVID-19 pandemic

- Patients with a low risk of developing liver dysfunction (preferably those with a Child-Pugh score <B8 and who do not have ascites, encephalopathy, or other cirrhosis-related complications).
- Assess the risks and benefits in patients aged >75 years and/or have comorbidities that increase the risk of developing severe forms of COVID-19.
- Consider using TAE to reduce the risk of complications associated with conventional TACE.
- The risks and benefits of performing TACE with SBRT and systemic treatment in patients at a high risk for severe SARS-CoV-2 infection, such as those aged >75 years and/or having comorbidities, and patients with multifocal tumors (>3 nodules), especially those who fall outside the Up-to-7 criteria (25), should be discussed by a multidisciplinary team.
- Patients with an unsatisfactory response to TACE during follow-up imaging examinations, especially after the second session, should be considered for early migration to systemic treatment to minimize the risk of liver decompensation and subsequent hospitalization.

### Treatment options during the COVID-19 pandemic

- Stereotactic body radiation therapy can be considered for patients who fall within the Up-to-7 criteria (25) and who have preserved liver function.
- Systemic therapy should be considered, particularly for patients with multifocal tumors (>3 nodules) who fall outside the Up-to-7 criteria (25).
- Strict monitoring with imaging can be carried out in selected cases.

### Systemic therapy for HCC during the COVID-19 pandemic

#### Treatment indications

- Systemic therapy should be initiated on the basis of the HCC management guidelines (17-20). This strategy is more commonly used for BCLC-C or BCLC-B tumors that show disease progression or for cases that have contraindications to locoregional modalities. In our institutional protocol, the standard first-line therapy for systemic treatment is sorafenib 400 mg PO BID. Other systemic therapies approved for HCC treatment have not been mentioned in this document since they are not available in the HC-FMUSP complex.
- Systemic treatment can be delayed in specific cases depending on the patient’s tumor burden, tumor growth rate, and liver function.
- For patients with treatment-naive BCLC-B HCC, migration to systemic treatment can be considered in cases of multinodular disease that have a low probability of achieving an objective response to TACE/TAE.
- Inclusion in clinical trials can be considered depending on the availability of active protocols and should be discussed individually with the patient.

### Recommendations during the COVID-19 pandemic

- Our institutional policy is to start sorafenib at a standard dose of 400 mg BID.
- In cases that have a high risk for adverse events (*e.g*., age >65 years, Child-Pugh A6-B7, relevant comorbidities, use of anticoagulants, use of antiretroviral therapy, arterial hypertension requiring two or more anti-hypertensive drugs), starting sorafenib at a reduced dose can be considered during the first 8 weeks to reduce the risk of early adverse events.
- Early recognition of adverse events should be taught to all patients to avoid unnecessary hospitalization.

○ Adverse events should be identified as early as possible to allow for prompt initiation of preventive/therapeutic measures; these include taking 2 mg of loperamide as needed, as well as using of moisturizing cream (10% urea, 2-3 times per day on the hands and feet) and sunscreen.

○ Online consultations should be conducted within 2-4 weeks after the patient started systemic treatment to aid in the early assessment and identification of adverse events.

### Treatment course

- In the absence of clinically relevant adverse events, treatment should be maintained at the current tolerated dose.
- For patients undergoing systemic treatment, consider performing remote visits every 4-6 weeks and laboratory tests (liver function and enzyme tests, kidney function tests, complete blood count, and alpha-fetoprotein levels) every 6-8 weeks.
- Patients who are using diuretics for ascitic-edematous decompensation and/or as secondary prophylaxis for hepatic encephalopathy should preferably be evaluated in face-to-face visits every 4 weeks.
- When a patient’s treatment requires adjustment, consultations should be conducted within 2 weeks online or personally at the discretion of the treating physician.
- Patients should be educated on warning signs such as diarrhea, fever, and dyspnea.
- Radiological assessment should be performed every 2-3 months; however, the interval can be increased to 3-4 months in the absence of symptoms/signs suggestive of tumor progression or liver dysfunction.
- In patients with suspected COVID-19, temporarily stopping systemic treatment is recommended until the infection status is confirmed (26). Systemic treatment can be resumed 14 days after the onset of symptoms and the patient has been afebrile for at least 4 days and does not have respiratory symptoms.

### Treatment options during the COVID-19 pandemic

- Stereotactic body radiation therapy can be used for patients with intermediate HCC who fall within the Up-to-7 criteria but have contraindications to TACE/TAE or are unresponsive to TACE/TAE (25). It may also be considered for patients who have advanced HCC with segmental tumoral portal vein thrombosis but without extrahepatic metastases.
- Strict monitoring with imaging can be carried out in selected cases.
- Exclusive palliative care can be considered.

### Follow-up after treatment during the COVID-19 Pandemic

Routine outpatient visits can be carried out online and face-to-face visits can be postponed. Laboratory and imaging tests should be performed to define the follow-up schedule.

- In case of tumor recurrence, a face-to-face consult should be conducted.
- In cases wherein HCC patients report symptoms suggestive of liver decompensation, such as ascites, confusion, and bleeding, or symptoms that may indicate tumor progression, a face-to-face consult should be scheduled.
- If a face-to-face consult is necessary, adopt individual protection and preventive measures as recommended by the World Health Organization and the Pan American Health Organization (4).
- Request new laboratory and imaging tests depending on the post-treatment follow-up protocol for the treatment performed and the time since the last therapy.
- In patients undergoing surgery or locoregional therapy, close follow-up monitoring should be carried out using imaging tests every 3-4 months in the first year and later every 6 months to monitor treatment response and possible tumor recurrence.
- In patients who exhibit complete response to treatment and who do not have evidence of recurrence for >2 years, imaging examinations can be postponed for up to 2 months.

## MANAGEMENT OF HCC PATIENTS WITH OR SUSPECTED OF HAVING COVID-19

- Rigorous monitoring of symptoms should be carried out to identify early signs suggestive of severe COVID-19 and other indications for hospital admission according to the institutional protocol.
- Patients undergoing systemic treatment should temporarily discontinue their medications. Treatment should resume after 14 days after the onset of symptoms and once the patient has been afebrile and free from respiratory symptoms for at least 3 days.
- Patients with HCC who are scheduled to receive locoregional or surgical treatment (resection/LT) but are suspected of having COVID-19 should have the treatment postponed until at least 14 days after the onset of symptoms and when fever or respiratory symptoms have been absent for at least 3 days.
- Patients being investigated for HCC who have indications for tumor biopsy but are confirmed or suspected of having COVID-19 should postpone the procedure until at least 14 days after the onset of symptoms and when fever or respiratory symptoms have been absent for at least 3 days.

## AUTHOR CONTRIBUTIONS

Chagas AL, da Fonseca LG, Coelho FF: designed, co-wrote, and drafted the manuscript

Saud LRC, Andraus W, Moreira AM, Abdala E: co-wrote and drafted the manuscript.

Menezes MR, Carnevale FC, Tani CM, Alencar RSSM: reviewed the manuscript

Albuquerque LAC, Herman P, Carrilho FJ: drafted and provided the manuscript version that received final approval

## References

[1] Guan WJ, Ni ZY, Hu Y, Liang WH, Ou CQ, He JX (2020). Clinical Characteristics of Coronavirus Disease 2019 in China. N Engl J Med.

[2] Johns Hopkins Coronavirus Resource Center https://coronavirus.jhu.edu/.

[3] Boletim Coronavírus Ministério da Saúde. https://covid.saude.gov.br/.

[4] Folha informativa COVID-19 Organização Mundial de Saúde (OMS) e Organização Pan-americana de Saúde (OPAS).

[5] ILCA Guidance for Management of HCC during COVID-19 Pandemic [April 8, 2020].

[6] Liang W, Guan W, Chen R, Wang W, Li J, Xu K (2020). Cancer patients in SARS-CoV-2 infection: a nationwide analysis in China. Lancet Oncol.

[7] Dai M, Liu D, Liu M, Zhou F, Li G, Chen Z (2020). Patients with cancer appear more vulnerable to SARS-CoV-2: A multicenter study during the COVID-19 outbreak. Cancer Discov.

[8] Desai A, Sachdeva S, Parekh T, Desai R (2020). COVID-19 and Cancer: Lessons from a Pooled Meta-Analysis. JCO Glob Oncol.

[9] Emami A, Javanmardi F, Pirbonyeh N, Akbari A (2020). Prevalence of Underlying Diseases in Hospitalized Patients with COVID-19: a Systematic Review and Meta-Analysis. Arch Acad Emerg Med.

[10] Deng G, Yin M, Chen X, Zeng F (2020). Clinical determinants for fatality of 44,672 patients with COVID-19. Crit Care.

[11] Mehta V, Goel S, Kabarriti R, Cole D, Goldfinger M, Acuna-Villaorduna A (2020). Case Fatality Rate of Cancer Patients With COVID-19 in a New York Hospital System. Cancer Discov.

[12] Zhang L, Zhu F, Xie L, Wang C, Wang J, Chen R (2020). Clinical characteristics of COVID-19-infected cancer patients: a retrospective case study in three hospitals within Wuhan, China. Ann Oncol.

[13] Kuderer NM, Choueiri TK, Shah DP, Shyr Y, Rubinstein SM, Rivera DR (2020). Clinical impact of COVID-19 on patients with cancer (CCC19): a cohort study. Lancet.

[14] Boettler T, Newsome PN, Mondelli MU, Maticic M, Cordero E, Cornberg M (2020). Care of patients with liver disease during the COVID-19 pandemic: EASL-ESCMID position paper. JHEP Rep.

[15] Couto CA, Pessoa MG, Ximenes RO (2020). Recomendações COVID American Association for the Study of Liver Diseases (AASLD). https://www.aasld.org/sites/default/files/2020-04/AASLD-COVID19-ClinicalInsights-PortVersion-April7.pdf.

[16] Brandão CE, Neto JMA Nota Técnica da Sociedade Brasileira de Hepatologia para COVID-19, 22/03/2020.

[17] Forner A, Reig M, Bruix J (2018). Hepatocellular Carcinoma. Lancet.

[18] European Association for the Study of the Liver (2018). EASL Clinical Practice Guidelines: Management of hepatocellular carcinoma. J Hepatol.

[19] Chagas AL, Mattos AA, Carrilho FJ, Bittencourt PL, Vezzozo DCP, Horvat N (2020). Brazilian Society of Hepatology updated Recommendations for Diagnosis and Treatment of Hepatocellular Carcinoma. Arq Gastroenterol.

[20] Heimbach JK, Kulik LM, Finn RS, Sirlin CB, Abecassis MM, Roberts LR (2018). AASLD guidelines for the treatment of hepatocellular carcinoma. Hepatology.

[21] Herman P, Perini MV, Coelho FF, Kruger JA, Lupinacci RM, Fonseca GM (2014). Laparoscopic resection of hepatocellular carcinoma: when, why, and how? A single-center experience. J Laparoendosc Adv Surg Tech A.

[22] Ramos RF, Lima DL, Benevenuto DS (2020). Recommendations of the Brazilian College of Surgeons for laparoscopic surgery during the COVID-19 pandemic. Rev Col Bras Cir.

[23] COVIDSurg Collaborative (2020). Global Guidance for Surgical Care During the COVID-19 Pandemic. Br J Surg.

[24] Lavarone M, Sangiovanni A, Carrafiello G, Rossi G, Lampertico P (2020). Management of hepatocellular carcinoma in the time of COVID-19. Ann Oncol.

[25] Mazzaferro V, Llovet JM, Miceli R, Bhoori S, Schiavo M, Mariani L (2009). Predicting survival after liver transplantation in patients with hepatocellular carcinoma beyond the Milan criteria: a retrospective, exploratory analysis. Lancet Oncol.

[26] Da Fonseca L, Carrilho FJ (2020). Can thrombotic events be a major concern in hepatocellular carcinoma patients under systemic treatment during SARS‐Cov‐2?. Liver Int.

