# Erratum for: Management of Hepatocellular Carcinoma during the COVID-19 Pandemic – São Paulo Clínicas Liver Cancer Group Multidisciplinary Consensus Statement

**DOI:** 10.6061/clinics/2021/e2192err

**Published:** 2021-02-01

CLINICS 2021;76:e2192err

Erratum for: doi: https://doi.org/10.6061/clinics/2020/e2192, published in 2020.

In the article Management of Hepatocellular Carcinoma during the COVID-19 Pandemic – São Paulo Clínicas Liver Cancer Group Multidisciplinary Consensus Statement

Replace “Edson Abadala” and “Chagas AL, da Fonseca LG, Coelho FF, Saud LRC, Abadala E, Andraus W, et al. Management of Hepatocellular Carcinoma during the COVID-19 Pandemic – Saõ Paulo Clínicas Liver Cancer Group Multidisciplinary Consensus Statement. Clinics. 2020;75:e2192” for:

“Edson Abdala” and “Chagas AL, da Fonseca LG, Coelho FF, Saud LRC, Abdala E, Andraus W, et al. Management of Hepatocellular Carcinoma during the COVID-19 Pandemic – Saõo Paulo Clínicas Liver Cancer Group Multidisciplinary Consensus Statement. Clinics. 2020;75:e2192”

